# A surgically salvaged case: emergency Root-Commando procedure for infective endocarditis and chronic dissecting aneurysm of the sinus of Valsalva

**DOI:** 10.1093/icvts/ivad207

**Published:** 2023-12-19

**Authors:** Yu Nosaka, Hiroki Kato, Hironari No

**Affiliations:** Department of Cardiovascular Surgery, Ishikawa Prefectural Central Hospital, Kanazawa, Japan; Department of Cardiovascular Surgery, Ishikawa Prefectural Central Hospital, Kanazawa, Japan; Department of Cardiovascular Surgery, Ishikawa Prefectural Central Hospital, Kanazawa, Japan

**Keywords:** Infective endocarditis, Dissecting aneurysm of the sinus of Valsalva, Root-Commando procedure

## Abstract

The Commando procedure for infective endocarditis is a high-risk intervention. However, infective endocarditis involving the intervalvular fibrosa is fatal in the absence of surgery. A 41-year-old man with no medical history visited a doctor with chest pain and dyspnoea. Ascending aortic dissection and vegetation on the mitral valve were noted on echocardiography, so he was transferred to our hospital. The diagnosis was dissecting aneurysm of the sinus of Valsalva and acute heart failure due to aortic regurgitation, mitral regurgitation and infective endocarditis. We decided on emergency surgery. Intraoperatively, we confirmed abscess extending to the left atrial roof and destruction of the intervalvular fibrosa, so we performed the Root-Commando procedure. The patient was saved and discharged 47 days after transfer to our hospital through the reoperation on postoperative day 30.

## INTRODUCTION

The Commando procedure for infective endocarditis (IE) is a high-risk procedure with a 1-year mortality rate of 20–30% [1]. While this is more risky in emergency cases, extensive IE involving the intervalvular fibrosa (IVF) has a mortality rate of 100% in the absence of surgical intervention [1].

## CASE REPORT

A 41-year-old man with no contributory medical history visited a local doctor with a 6-day history of chest pain and dyspnoea. He was admitted to the hospital with acute heart failure. The next day, ascending aortic dissection and vegetation on the mitral valve were noted on echocardiography, so he was transferred to our hospital.

**Figure 1:**
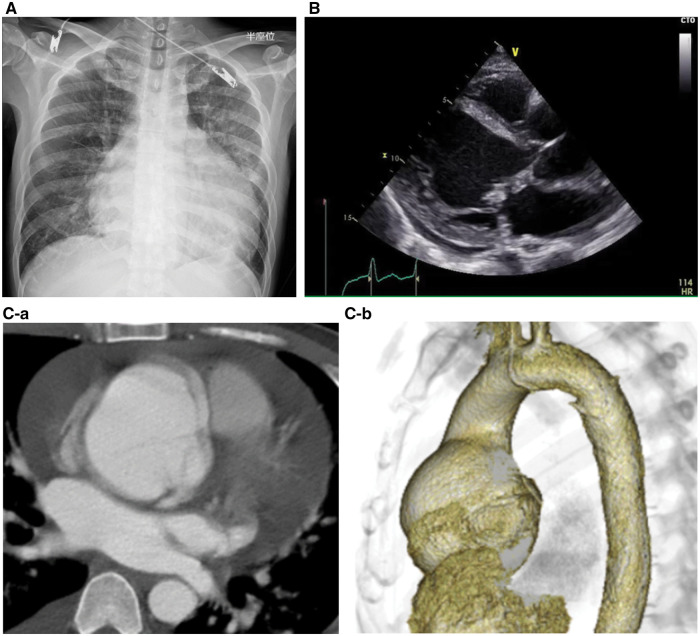
(**A**) Preoperative chest X-ray. (**B**) Vegetation on the mitral valve by echocardiography. (**C-a** and **C-b**) Dissecting aneurysm of the sinus of Valsalva by computed tomography.

He showed poor oxygenation (oxygen saturation, 93% under oxygen by mask at 8 l/min). Laboratory investigations revealed congestive heart failure (brain natriuretic peptide, 595.7 pg/ml), congestive liver injury (aspartate aminotransferase, 6251 U/l; alanine aminotransferase, 1976 U/l), severe inflammation (C-reactive protein, 37.3 mg/dl) and hyperglycaemia (haemoglobin A1c, 13.6%). Chest X-ray showed pulmonary congestion and cardiac enlargement (cardiothoracic ratio, 69.1%) (Fig. 1A). Echocardiography showed severe aortic regurgitation, mitral regurgitation (Video 1) and vegetation on the mitral valve (Fig. 1B). Computed tomography (CT) showed dissecting aneurysm of the sinus of Valsalva (78 mm) (Fig. 1C-a and C-b). The diagnosis was dissecting aneurysm of the sinus of Valsalva and acute heart failure due to aortic regurgitation, mitral regurgitation and IE. We decided to perform emergency surgery.

Incision of the pericardium released a large amount of serous pericardial fluid (Video 2). Dissection of the sinus of Valsalva was considered chronic. The abscess extended to the left atrial roof, wall of the left ventricle, anterior mitral leaflet and IVF. We therefore decided to perform the Root-Commando procedure (not reconstructing his mitral leaflet) for enough debriding vegetation considering his severe diabetes. The aortic valve was removed, creating coronary buttons, and the sinus of Valsalva and basal tissues were resected. The anterior mitral valve and IVF were subsequently resected. After debridement of the infected tissue, a mechanical valve (29-mm; St Jude Medical, Abbott Medical Japan, Tokyo, Japan) was implanted supra-annularly, placed along the posterior mitral annulus. A double patch of bovine pericardium was applied. One side was used for reconstruction of the left atrial roof. The IVF was reconstructed using a folded double patch. We made a composite graft comprising a Valsalva graft (28-mm; Gelweave Valsalva, TERUMO, Tokyo, Japan) and a mechanical valve (25-mm St Jude Medical). The hand-made mechanical valved conduit leaving free margin of graft below the valve was a modification (proposed by Urbanski *et al.* [2]) to implant in the fragile. The composite graft was sewn to the reconstructed autologous IVF and aortic annulus (left and right coronary cusp). The left and right coronary buttons were anastomosed to the composite graft. Finally, the ascending aorta was anastomosed to the graft. Although bleeding was difficult to control, the surgery was successfully completed.

**Figure 2:**
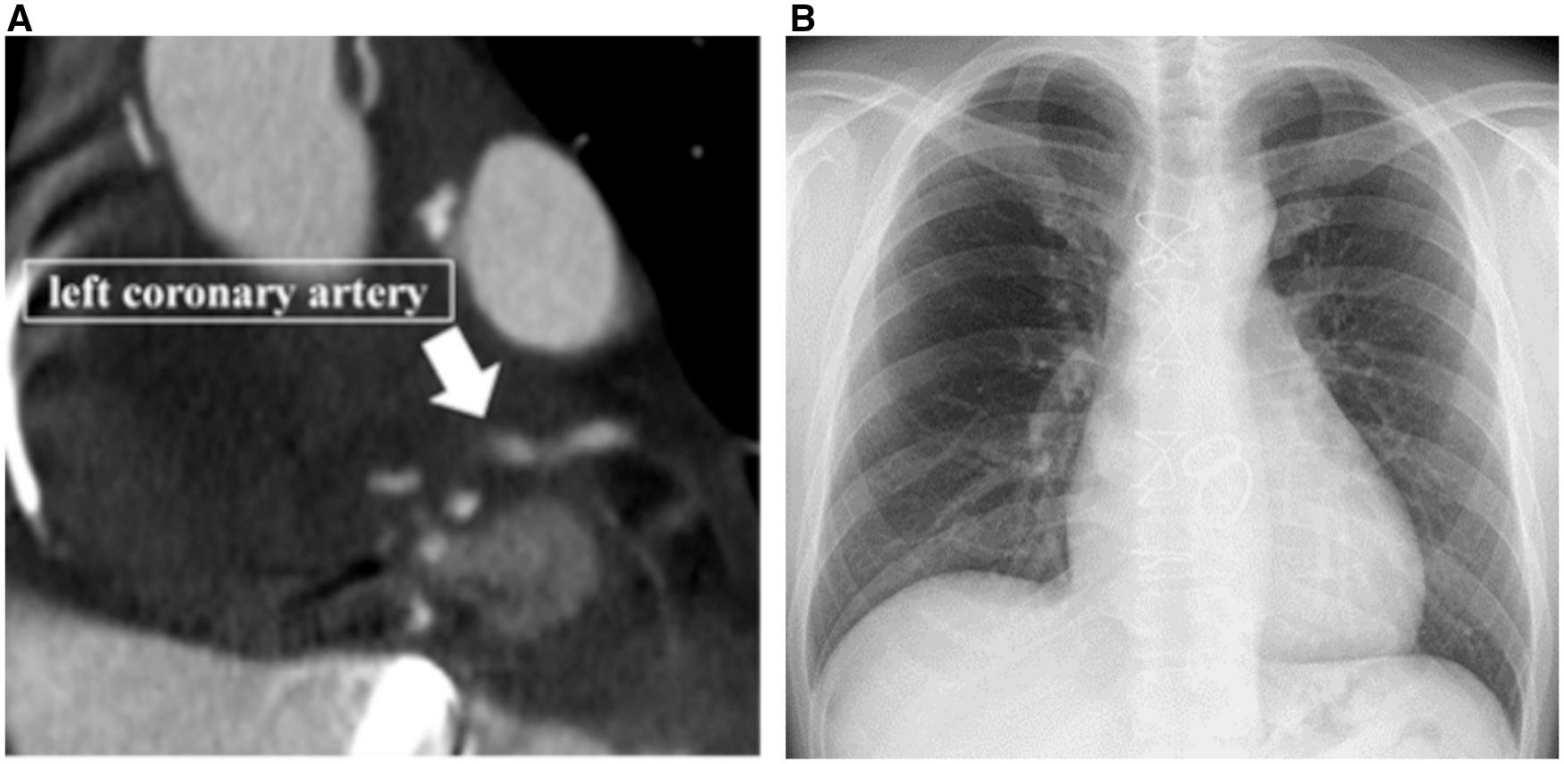
(**A**) Pseudoaneurysm under the left coronary button. (**B**) Postoperative chest X-ray.

Postoperative course was good, but he had back pain on postoperative day 30. CT showed pseudoaneurysm under the left coronary button (Fig. 2A), so we performed reoperation. The coronary button was removed and patched by bovine pericardium. Left coronary artery was reconstructed with an artificial graft (PROPATEN Vascular Graft 8 mm; GORE, Tokyo, Japan) to the composite graft like Piehler method. After reoperation, chest X-ray showed improvement of cardiac enlargement (cardiothoracic ratio, 48.7%), while CT shows good flow in the artificial graft and coronary artery (Fig. 2B and Video 3). The patient was therefore discharged 47 days after admission to our hospital.

## DISCUSSION

Despite the high-risk of the Commando procedure for IE, the subsequent rates of reintervention (9.1% within 5–10 years) and IE recurrence (15% for 10 years) are satisfactory [3]. However, the reoperation rate for bleeding is 13.1%, which might be related to the emergency setting, fragility of infected tissue and difficulty achieving hemostasis due to the inaccessibility of posterior suture lines [4]. The present procedure involved reoperation for posterior bleeding (pseudoaneurysm). The case has many high-risk factors: infected tissue, inflammation due to the dissection and emergency surgery. Reducing the risk of postoperative bleeding is important in the Commando procedure. Some surgeons have suggested modifications. Chen *et al.* [4] suggested extended transseptal approach for better exposure and using extra bovine pericardium patch for repair of the right atrium to reduce tension on the IVF and transfer the suture line from the posterior to the anterior aspect. Uchida *et al.* [5] suggested in which supra-annular aortic valve replacement with a Solo Smart bovine pericardial stentless valve was employed instead of aortic root replacement. This modification is expected to render complex reconstructive surgery technically less demanding. We need to research better methods for modified Commando procedures that could be used in emergency surgeries.

## CONCLUSION

We performed an emergency Root-Commando procedure for IE and dissecting aneurysm of the sinus of Valsalva against a background of type 1 diabetes. We could salvage the patient through the reoperation for pseudoaneurysm of the left coronary button.

## Data Availability

All relevant data are within the manuscript and its Supporting Information files.
